# Prostatic abscess due to carbapenem-resistant *K. pneumonia*: A case report

**DOI:** 10.1097/MD.0000000000036380

**Published:** 2023-12-08

**Authors:** Wen-Qiang Zhou, Zhi Chen, Xue-Ju Cai

**Affiliations:** a Medical Department of Graduate School Nanchang University, Nanchang, Jiangxi Province, China; b Jiangxi Provincial People’s Hospital, The First Affiliated Hospital of Nanchang Medical College, Nanchang, Jiangxi Province, China; c Department of Anesthesiology, Jiangxi Provincial People's Hospital, The First Affiliated Hospital of Nanchang Medical College, Nanchang, Jiangxi Province, China.

**Keywords:** carbapenem-resistant *K. pneumoniae*, case report, omadacycline, prostatic abscess, *K. pneumoniae*

## Abstract

**Rationale:**

Due to the widespread use of broad-spectrum antibiotics, the morbidity of prostate abscesses (PA) has declined dramatically. However, under special circumstances, such as invasive procedures and immunosuppressive conditions, some patients are more likely to develop this disease. Here, we present the case of a 21-year-old man, diagnosed with PA, with a history of chronic steroid use and a long-term indwelling urinary catheter. The pathogen was confirmed as carbapenem-resistant *Klebsiella pneumoniae*, a rare bacterium. This case indicates that immunodeficiency and invasive catheter use may be risk factors for PA and opportunistic bacterial infections.

**Patient concerns:**

A 21-year-old young man presented with sudden onset of high fever (39.7°C). The patient had a history of long-term use of steroids and long-term indwelling urinary catheter. Digital rectal examination revealed obvious swelling and tenderness of the prostate. Subsequent pelvic magnetic resonance imaging showed a high signal lesion measuring 2.1 × 2.9 × 2.8 cm with T1 enhancement and T2 enhancement.

**Diagnoses:**

On the 8th day of hospitalization, the patient underwent a PA drainage procedure and a pus culture was conducted. Subsequent pus and urine cultures showed the presence of *Klebsiella pneumoniae*, which exhibited resistance to all injectable carbapenems, cephalosporins, aminoglycosides, piperacillin-tazobactam, and quinolone drugs.

**Interventions:**

On the 8th day of hospitalization, the patient underwent PA drainage surgery under general anesthesia to drain the abscess and relieve obstruction. After the surgery, the patient received a 2-week treatment of doxycycline.

**Outcomes:**

Finally, the patient was discharged after recovery and did not experience recurrence during the 6-month follow-up period.

**Lessons:**

PA is not commonly found, but some patients are more susceptible to this disease under certain host conditions. Immunodeficiency and invasive catheter use may be risk factors for PA and opportunistic bacterial infections. The use of omadacycline for the treatment of carbapenem-resistant *Klebsiella pneumoniae* infections appears to be effective.

## 1. Introduction

Since the invention of broad-spectrum antibiotics, the incidence of prostatic abscesses has decreased significantly.^[1]^ Overall, the annual incidence of prostatic abscesses (PA) is 0.5% of all urological diseases, with mortality ranging from 1% to 16%.^[2]^ Due to the rarity of its occurrence, no official guidelines for diagnosis and treatment have been developed.

Before the widespread use of antibiotics, *Neisseria gonorrhoeae* was the predominant pathogen (75%), but today gram-negative bacteria predominate.^[1,3]^ Gram-negative *Klebsiella pneumoniae (KP*) commonly causes PA; however, its prevalence varies by geographic region. In southern Taiwan, *KP* is the predominant pathogen of PA,^[4]^ whereas in South Korea, *KP* (17.3%) is the second most dominant bacterium, after *E. coli* (40.4%).^[5]^ The incidence of *KP*-induced PA seems to be increasing, and related reports are also increasing. However, the pathogens are all carbapenem-sensitive *KP*, and there are no related reports of PA caused by carbapenem-resistant *Klebsiella pneumoniae* (CR*KP*). Here, we report a case of PA induced by CR*KP*.

## 2. Case report

A 21-year-old presented to the emergency department with a sudden onset of high fever (39.7°C), which decreased after self-administration of antipyretics. The patient experienced lower urinary tract symptoms such as frequency and urgency in the past month. Over 3 months previously, the patient was diagnosed with autoimmune encephalomyelitis and had a long-term indwelling catheter placed and long-term hormone treatment. After discharge, they continued to take prednisone acetate tablets 50 mg/day for more than 1 month.

**Figure 1.**
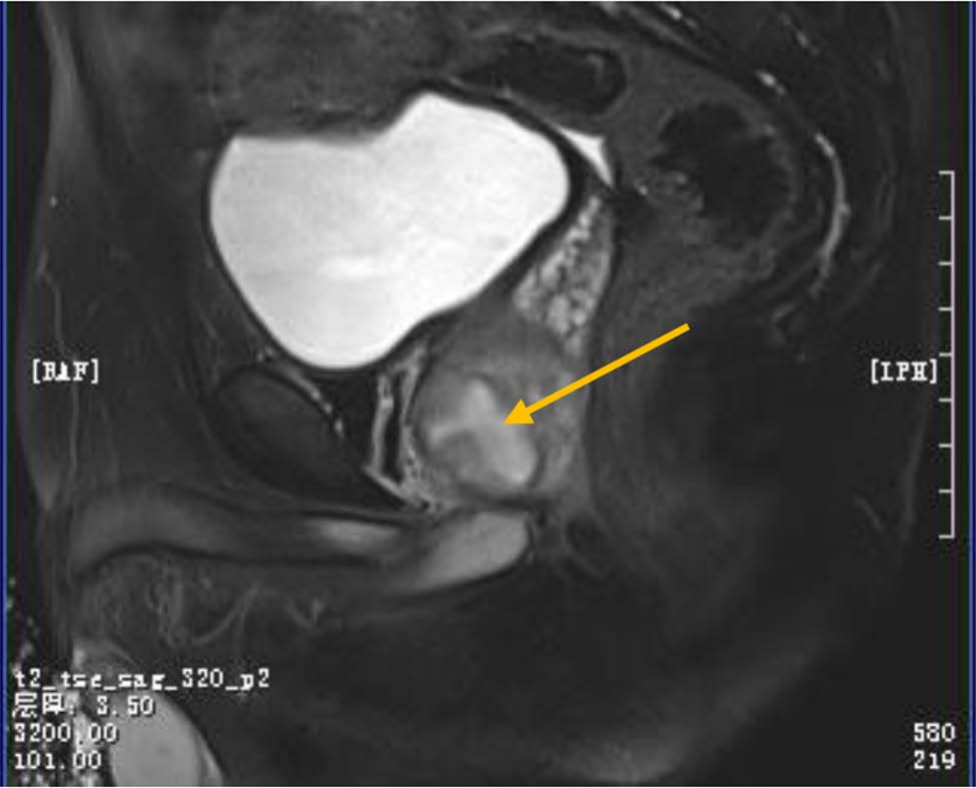
Magnetic resonance imaging of the prostate showed a prostate abscess.

On this admission, the patient was conscious, with a blood pressure of 125/82 mm Hg, body temperature of 37°C, and heart rate of 98 beats/min. Anal palpation revealed a markedly elevated and tender prostate. Laboratory tests suggest an inflammatory reaction (Table 1). The subsequent pelvic magnetic resonance imaging showed a 2.1 × 2.9 × 2.8 cm hyperintense lesion on T1 and hyperintense T2, which was diagnosed as prostatic abscess (Fig. 1).

**Table 1:** Laboratory results.

Laboratory parameters (reference range)	On admission	1st after surgery	3st after surgery	6st after surgery	9st after surgery	12st after surgery	16st after surgery	21st after surgery
Leukocyte count (3.5–9.5 × 10^9^/L)	9.04	11.71	9.13	20.07	13.29	10.32	8.75	7.38
Neutrophil count (1.4–7.12 × 10^9^/L)	6.77	9.21	6.1	16.78	10.74	7.88	6.12	5.18
Neutrophil percentage (40%–75%)	74.90%	78.70%	66.80%	83.70%	80.90%	76.40%	73.00%	70.20%
CRP (0–8 mg/L)	37.8	142	15.8	7.65	37.1	23.2	15.3	7.3
PCT (<0.5 ng/ml)	0.57	3.26	0.23	6.28	0.12	0.08	0.02	0.03
Urine leukocyte count (0–15/μL)	26.0	20.5	29.4	29.4	148.1	17.5	57.2	9.5

CRP = C-reactive protein, PCT = procalcitonin.

On the 8th day of admission, PA drainage was performed under general anesthesia to drain the abscess, relieve obstruction, and perform pus culture. That evening, the patient developed a high fever (38.9°C), chills, and hypotension (88/62 mm Hg). Laboratory tests showed elevated inflammatory markers (Table 1). We considered septic shock caused by postoperative hematogenous infection and empirically used imipenem for anti-infective treatment.

After 5 days of treatment, the patient still had recurrent low-grade fever without a significant decrease in inflammatory markers. Subsequent culture of urine and pus yielded *KP*, which was resistant to all injectable cephalosporins, aminoglycosides, carbapenems, piperacillin-tazobactam, and quinolones. Minocycline, tigecycline, and polymyxin were all potentially effective (Table 2). We then switched treatment to omalocycline. After 2 weeks of omalocycline treatment, the patient’s temperature returned to normal and the symptoms of urinary frequency and urgency completely resolved. Finally, the patient was discharged after recovery and did not experience recurrence during the 6-month follow-up period.

**Table 2:** Antimicrobial susceptibility.

Antibiotics	MIC/KB	Sensitivity
Nitrofurantoin	≥128	R
Gentamicin	≥16	R
Ampicillin	≥32	R
Cefuroxime	≥32	R
Ceftazidime	≥32	R
Cefepime	≥32	R
Cefoxitin	≥32	R
Ceftriaxone	≥64	R
Piperacillin/tazobactam	≥128/4	R
Ampicillin/sulbactam	≥64/32	R
Meropenem	≥16	R
Imipenem	4	R
Compound sulfamethoxazole	≥64	R
Levofloxacin	≥8	R
Ciprofloxacin	≥4	R
Cefoperazone/ Sulbactam	≥64/32	R
Minocycline	≤4	S
Polymyxin B	0.5	M
Tigecycline	≤4	S
Ceftolozane/tazobactam	≥8/152	R

M = moderate, R = resistant, S = susceptible.

## 3. Discussion

A prostatic abscess is an uncommon infection that is usually a complication of an ascending genitourinary infection caused by acute bacterial prostatitis or gram-negative bacilli in the intestine. Gram-negative bacteria are the predominant pathogens. Among these, *KP* is an important pathogen. The incidence of *KP*-induced PA seems to be increasing, and related reports are also increasing.^[1,3]^ However, there are no related reports on PA caused by CR*KP*. To our knowledge, this is the first reported case of CR*KP*-induced PA.

The incidence of prostatic abscesses is low. However, under special circumstances, such as invasive procedures and immunosuppressed states, some patients may develop this disease. Risk factors for PA include diabetes mellitus, human immunodeficiency virus infection, prostatitis, urinary tract devices, indwelling catheters, voiding dysfunction secondary to obstruction, and neurogenic bladder.^[6]^ Our case concurs that immunosuppression and long-term indwelling catheter use are risk factors for PA, and *KP* bacteremia may be a new risk factor for PA.

*KP* is ubiquitous in nature and is one of the most important opportunistic pathogens, causing a variety of human infections such as bloodstream infections, urinary tract infections, surgical site infections, and pneumonia.^[7]^ Carbapenem-resistant strains are rapidly increasing owing to the spread of resistant plasmids and high-risk clones. Between 2001 and 2011, the prevalence of CR*KP* strains associated with central-line bloodstream infections in the United States increased from 1.6% to 10.4%.^[8]^ In 2016, the prevalence of CR*KP* in different provinces of China ranged from 0.9% to 23.6%, with an average of 8.7%.^[9]^ As antimicrobial resistance is increasing, various aspects of modern medicine are under threat, including oncology and surgical care. Additionally, long-term use of antimicrobial agents is widely accepted to treat CR*KP*. Long-term antibiotic treatment not only increases the treatment cost for patients but also increases the incidence of multi-drug resistant bacteria, which poses a dilemma for healthcare systems.

The incidence of CR*KP* in hospitalized patients is associated with immunosuppression, length of intensive care unit stay, severity of underlying disease, and previous exposure to different classes of antibiotics, including carbapenems, fluoroquinolones, cephalosporins, and glycopeptides.^[10]^ Our case showed that immunosuppression, recent surgery, and previous exposure to different classes of antibiotics are risk factors for CR*KP* acquisition. The independent risk factors for CR*KP* exposure were prolonged hospital stay and sharing a room with a known carrier. Screening for CR*KP* at admission would protect against CR*KP* infection.^[11]^ Thus, strict infection control is essential to prevent the spread of resistance.

*KP* is known for its multidrug resistance due to its ability to acquire resistance to different classes of antibiotics, including carbapenems, with limited availability of effective drugs. In fact, appropriate antibiotic treatment for CR*KP* remains an open question in clinical practice. In this case, we used omadacycline to treat CR*KP* bloodstream infection, the treatment effect was satisfactory, and no significant complications were noted.

Omadacycline, a tetracyclic antibacterial drug, is a novel aminomethylcycline antibiotic. It has a broad spectrum of antimicrobial activity against gram-positive and gram-negative bacteria, shows good antimicrobial activity against *KP*,^[12]^ and shows activity against 2 of the most common tetracycline resistance mechanisms, namely ribosome protection and active efflux.^[13]^ In addition, omadacycline has a lower plasma protein binding rate than tigecycline and a larger oral distribution volume than other tetracyclines, suggesting that omadacycline has higher tissue permeability and has advantages in the treatment of systemic infections.^[14]^ Finally, its availability as an intravenous or oral formulation, long serum half-life, and once-daily administration are benefits of this next-generation tetracycline. It is worth noting that omadacycline is more cost-effective than tigecycline and polymyxin in China. Therefore, considering the efficacy and economic benefits of omadacycline, we believe that this is a better choice.

## 4. Conclusion

PA is not commonly found, but some patients are more susceptible to this disease under certain host conditions. Immunodeficiency and invasive catheter use may be risk factors for PA and opportunistic bacterial infections. The use of omadacycline for the treatment of CR*KP* infections appears to be effective.

## Acknowledgments

The authors would like to thank the patient and all the doctors and nurses participated in the case.

## Author contributions

**Conceptualization:** Wen-Qiang Zhou.

**Data curation:** Wen-Qiang Zhou.

**Funding acquisition:** Zhi Chen.

**Investigation:** Wen-Qiang Zhou.

**Methodology:** Wen-Qiang Zhou.

**Project administration:** Xue-Ju Cai.

**Resources:** Zhi Chen.

**Supervision:** Zhi Chen.

**Validation:** Zhi Chen, Xue-Ju Cai.

**Writing – original draft:** Wen-Qiang Zhou.

**Writing – review & editing:** Zhi Chen, Xue-Ju Cai.
